# Gaps between normative advancement and clinical response: the inclusion of the deaf community in chilean public health

**DOI:** 10.17843/rpmesp.2026.432.15099

**Published:** 2026-06-25

**Authors:** Valeria Campos, Irene Villalobos-Saldivia, Sebastián Quiñones

**Affiliations:** 1 Departamento de Salud Pública, Facultad de Medicina, Universidad San Sebastián, Sede Tres Pascualas, Octava, Chile.; 2 Facultad de Ciencias Sociales, Escuela de Psicología, Universidad Santo Tomás, Santiago, Chile.; 3 Universidad de Santiago de Chile, Santiago de Chile; Chile.

**Keywords:** Deafness, Persons with Disabilities, Health Systems, Health Legislation, Human Rights, Sign Language

## Abstract

Chile has experienced a progressive evolution of its regulatory framework toward a rights-based approach; however, the exclusion of the Deaf community in the health system persists. This research analyzes the political-institutional gaps between the regulatory framework of rights and clinical practices in the Chilean health system. The findings evidence a structural decoupling linked to institutional audism, which reduces Sign Language to an instrumental resource and prioritizes technocratic solutions of remote interpretation over in-person practices of cultural and communicative competence. Furthermore, judicialization has contributed to legitimizing the outsourcing of guarantees to the municipal level, deepening territorial inequities and worsening access, especially in high-complexity care. It is concluded that linguistic rights cannot depend on administrative wills and require a political-institutional reorientation with the structural incorporation of intercultural facilitators into the health network.

## INTRODUCTION

Historically, the understanding of deafness has transitioned from a pathological view towards a human rights approach. Under the traditional medical-rehabilitative paradigm, the deaf person was considered a «body with a deficit» that required correction to integrate into the hearing norm. This perspective, called audism, operates as a structure of discrimination that values the ability to hear and speak over visual and gestural experience, ignoring the psychosocial dimension of deafness ^1^^-^^3^. However, the emergence of the social model of disability and deaf studies (*Deaf Studies)* have redefined this condition not as a deficiency, but as an experience of human diversity that gives rise to a community with its own identity, history, and language through sign language (SL) ^2^^,^^3^.

From public health, this paradigm shift is decisive because it positions the deaf community as a linguistic minority whose access depends on the adequacy of healthcare environments. The sociocultural approach and deaf studies have consolidated this perspective by recognizing a community with its own identity and culture, sustained by the use of SL, in contrast to the medical model ^2^^,^^3^. In articulation with the disability discourse, this has favored the recognition of rights and the demand for adjustments to guarantee equitable access to health, education, and other services ^2^^,^^3^. However, globally, close to 70 million deaf people continue to face barriers in health, mainly due to communication difficulties ^4^. Evidence shows that this gap operates as a social determinant of health, increasing the risk of erroneous diagnoses, weakening therapeutic adherence, violating informed consent, and being associated with worse health outcomes ^4^. Therefore, linguistic access constitutes an ethical and human rights imperative.

In Chile, it is estimated that more than 500 thousand deaf people or people with hearing disabilities live, who continue to face access limitations and persistent forms of exclusion due to the insufficiency of state policies that effectively incorporate the sociocultural approach ^4^. Although normative advances and the ratification of international treaties have established explicit obligations to protect their linguistic and cultural rights, including linguistic autonomy recognized in recent laws such as Law N.º 21.303 ^4^, these frameworks have not consistently translated into sustained transformations within the public health institutionality. In this context, the growing judicialization of health care expresses the gap between legal recognition and health practice, and suggests the persistence of audist dynamics that hinder the effective incorporation of SL and perpetuate exclusion ^5^^-^^8^.

Given this persistent disconnection, it is urgent to examine the structural barriers that hinder the translation of legal mandates into effective practices and devices within the health system. In this regard, this study analyzes the political-institutional gaps between the normative framework of rights and clinical practices in Chile, identifying the historical and current factors that restrict the recognition of the Deaf community as a subject of health rights.

## METHODOLOGICAL APPROACH

The study adopted a qualitative approach ^9^ and was developed between 2024 and 2025. A normative documentary analysis ^10^ was carried out, understood as a systematic and comparative review of legal and technical texts aimed at extracting mandates, definitions, standards, and enforceable procedures from the regulatory framework of the health sector in Chile. The corpus included laws, decrees, regulations, technical standards, protocols, ministerial guidelines, and ratified international treaties.

The search covered scientific and grey literature published between 1950 and 2024. Indexed databases (PubMed, Web of Science, SciELO, and Google Scholar) and repositories of international organizations (United Nations Organization [UN], World Health Organization [WHO], Organization of American States [OAS]) and national ones (National Congress Library, Ministry of Health of Chile [MINSAL], National Disability Service [SENADIS]) were consulted. The strategy combined DeCS/MeSH descriptors and free terms in Spanish and English, also incorporating historical denominations to reconstruct the normative evolution.

The objective of the research was to analyze the political-institutional gaps between the regulatory framework of rights and the clinical practices of the Chilean health system, identifying historical and current barriers that limit the recognition of the Deaf community as a subject of health rights. In order to operationalize this purpose and guide the analysis, two specific objectives were defined: to identify and characterize the themes, approaches, and definitions present in the health regulatory frameworks referring to the Deaf community, considering their historical evolution.

The document selection and analysis process was organized into six phases, with the research team participating in each stage through collaborative work, deliberation, and control of coding and synthesis, seeking to strengthen analytical consistency and methodological rigor ^10^. The phases included: (a) definition of purpose and scope; (b) establishment of inclusion and exclusion criteria; (c) search design and execution; (d) corpus formation, registration, and organization; (e) thematic categorization; and (f) synthesis and integration of results. Throughout the procedure, ethical guidelines inherent to qualitative research were safeguarded ^10^.

**Figure 1:**
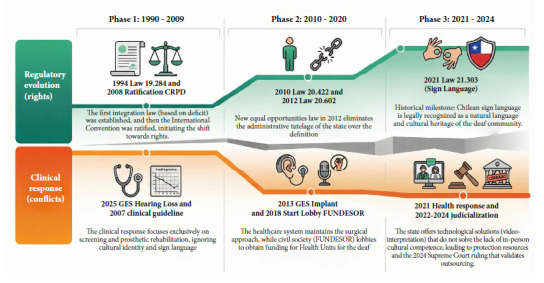
Comparative timeline between the regulatory evolution of rights and the clinical response of the Chilean health system (1990-2024).

Source: Own elaboration based on documentary analysis. The graphic design of this figure was assisted by generative artificial intelligence tools (NotebookLM/ Gemini), under supervision and content validation by the authors.

Subsequently, the documents were chronologically ordered and coded in ATLAS.ti; this strategy allowed systematizing the corpus, reconstructing its historical evolution, and establishing thematic categories with their respective analytical codes. The final corpus included 26 documents from the 1982-2023 period: five official international instruments (table 1), mainly from the UN, and national documents (tables 2 and 3) corresponding to laws, clinical guidelines, public policies, judicial rulings, hearings, decrees, a draft resolution, and an agreement. For the visualization of the synthesis of results (figure 1). Additionally, generative artificial intelligence tools were used exclusively for graphic design purposes, using the structured and validated database by the research team. Together, the procedure consolidated a systematized, traceable, and suitable documentary corpus for normative interpretation.

**Table 1:** Evolution of international instruments on disability and rights of the deaf community (1982-2017).

Title	Entity	Year	Purpose and Key contents	Legal nature
World Programme of Action Concerning Disabled Persons ^11^	UN	1982	To promote effective measures for the prevention of disability, rehabilitation and the achievement of the objectives of 'full participation' of persons with disabilities in social life and development and of 'equality'.	Non-binding
Standard Rules on the Equality of Opportunities for Persons with Disabilities ^12^	UN	1993	Ensure that girls, boys, women, and men with disabilities, in their capacity as members of their respective societies, can exercise the same rights and obligations as others. Defines state supervision mechanisms.	Non-binding
Inter-American Convention for the Elimination of all Forms of Discrimination against Persons with Disabilities ^13^	OAS	1999	Prevent and eliminate all forms of discrimination against persons with disabilities and promote their full integration into society. It provides broader regulation than previous general instruments.	Binding
Convention on the Rights of Persons with Disabilities ^14^	UN	2008	Promote, protect and ensure the full enjoyment and on an equal basis of all human rights and fundamental freedoms. It marks the paradigm shift by explicitly recognizing the cultural and linguistic identity of deaf people.	Binding
International Day of the Sign Languages ^15^	UN	2017	Raise awareness about the importance of sign languages for the full realization of human rights of deaf people. Resolution A/72/439.	Non-binding

UN: United Nations Organization, OAS: Organization of American States.

**Table 2:** Regulatory and technical trajectory: laws, clinical guidelines, and public health policies (1994-2024).

Year	Document/title	Institution	Purpose and content	Type of Instrument
1994	Law N.º 19.284 ^16^	Ministry of Planning and Cooperation	Establish the form and conditions that allow for obtaining the full integration of persons with disabilities in society, and ensure the full exercise of the rights that the Constitution and laws recognize.	Law
2002	Decree 99 ^17^	Ministry of Foreign Affairs	Approve and promulgate the Inter-American Convention on the Elimination of All Forms of Discrimination against Persons with Disabilities in Chile.	Decree
2005	Clinical guideline GES: Sensorineural hearing loss of the premature infant ^18^	Ministry of Health, National Health Fund, National Board for School Aid and Scholarships, and National Disability Fund	Create a national program for early detection of hearing loss in very low birth weight preterm infants, establishing guidelines for screening, diagnosis, treatment, and rehabilitation.	Clinical Guideline
2007	Clinical guideline: Hearing loss in people aged 65 years and older ^19^	Ministry of Health	Improve quality of life and reduce morbidity and mortality in older adults with bilateral hearing loss, optimize the prescription and fitting of hearing aids, and increase adherence to their use.	Clinical guideline
2008	Clinical guideline: Cochlear implant ^20^	Ministry of Health	Provide recommendations for the rehabilitation of cochlear implant candidates, optimizing the process in the health network and facilitating the professional team's decision-making.	Clinical guideline
2008	Decree 201 ^21^	Ministry of Foreign Affairs	Approve and enact the Convention on the Rights of Persons with Disabilities (CRPD) and its Optional Protocol.	Decree
2010	Law N.º 20.422 ^22^	Ministry of Planning	Guaranteeing equality of opportunity and social inclusion, ensuring the full enjoyment of rights and eliminating discrimination.	Law
2012	Law N.º 20.602 ^23^	Ministry of Social Development; Undersecretary of Social Services	Repeal the sixth paragraph of the first transitory article of Law 20.422, which established that «The State, together with the community... will define, within a period of three years, Chilean sign language».	Law
2013	AUGE Clinical Guideline: Hearing loss in children under 2 years old ^24^	Ministry of Health	Provide a comprehensive approach for early detection, diagnosis, and treatment. Includes use of hearing aids or implants and speech therapy to improve communicative development.	Clinical guideline
2013	National Policy for Social Inclusion 2013-2020 ^25^	National Disability Service, Ministry of Social Development	Foster the respect for rights and generate access to inclusive and specialized health services, focusing on prevention, treatment, habilitation, and rehabilitation.	Public policy
2016	Law N.º 20.927 ^26^	Ministry of Social Development	Ensure access to information on TV, forcing channels to use subtitles and sign language in important programs such as emergency news and public campaigns.	Law
2019	Clinical Guideline: Hearing Loss in Children Under 4 Years ^27^	Ministry of Health	Recommendations for detection and treatment, focusing on optimizing hearing aids and speech therapy to ensure social inclusion.	Clinical guideline
2020	Salud Responde Program (Implementation) ^28^	Ministry of Health	Incorporation of accessible guidance in emergency situations and administrative consultations through inclusive channels.	Public policy
2021	Law N.º 21.303 ^29^	Ministry of Social Development and Family	Promote the use of Chilean sign language, ensuring access to information and services. It recognizes LS as cultural heritage and natural language.	Law
2022	National Auditory Health Plan 2021-2030 ^30^	Ministry of Health	Improve hearing health and prevent hearing loss through early diagnosis, treatment, and rehabilitation, promoting social inclusion.	Public policy
2024	Health Responds Program (Video-interpretation) ^31^	Ministry of Health	Incorporation of Chilean Sign Language for health care remotely via video.	Public policy

**Table 3:** Social mobilization and judicialization: implementation gap and citizen response (2017-2024).

Year	Milestone/Action	Institutional actor involved	Description of the conflict/demand
2017	Audience of Lobby ^32^	MINSAL (DIVAP)	Fundación Sordos Chilenos requests funding for USS from Soledad Turra.
2017	Audience of Lobby ^33^	Undersecretary of Public Health	Fundación Sordos Chilenos requests funding for USS from Bernardo Martorell.
2017	Resolution Exempt No. 3119 ^34^	SS Metropolitano Norte / Muni. Recoleta	Local agreement for USS project.
2018	Opening USS The Forest ^35^	Muni. El Bosque / CESFAM Santa Laura	Opening of the first USS, municipal management without central structural funding.
2019	Audience of Lobby ^36^	MINSAL (DIVAP)	Fundación Sordos, Federación Nacional, and CESFAM Santa Laura insist on USS funding.
2022	Draft Resolution No. 1178 ^37^	Chamber of Deputies	The Legislature requests the Executive branch to mandate staff trained in sign language in public health.
2022	Lobby Hearing ^38^	Undersecretary of Public Health	Fundación Sordos and Federación de Intérpretes request USS funding from Undersecretary Cuadrado.
2022	Lobby Hearing ^39^	Healthcare Networks / DIVAP	Request for a joint working group to address healthcare access through a community proposal.
2023	Ruling Case 66.839-2022 ^5^	Court of Appeals of Concepcion	Orders a permanent interpreter at Hospital Grant Benavente (infant case).
2023	Role Failure 100.326-2022 ^6^	Court of Appeals of Concepcion	Upholds appeal against CESFAM Víctor M. Fernández for neglect a deaf pregnant patient.
2024	Role Failure 4070-2023 ^7^	Court of Appeals of San Miguel	Orders Hospital Barros Luco to guarantee an interpreter for an oncology patient.
2024	Revocation Role 9.186-2024 ^8^	Supreme Court	Revokes previous ruling; validates external coordination with CESFAM Santa Laura (municipal) for interpretation.

MINSAL: Ministry of Health of Chile, USS: Health Unit for Deaf People, DIVAP: Primary Care Division, CESFAM: Family Health Center.

## RESULTS

The documentary analysis evidences a slow and conflicting transition of paradigms in disability, characterized by a structural gap between normative progress and clinical response that is chronologically summarized in figure 1.

### Hegemony of the biomedical model and the beginning of normative tension (1982-2010)

Internationally, although foundational instruments such as the World Programme of Action ^11^ and the UN Standard Rules ^12^ incorporated general concepts of equality, they did not explicitly recognize deaf culture and maintained an undifferentiated notion of disability. Furthermore, they lacked binding force.

In the regional scope, Chile took an important legal step by ratifying in 2002 Decree 99 ^17^, which promulgated the Inter-American Convention (CIADIS) ^13^. Although this was the first binding instrument, its text focused on the prevention and elimination of discrimination, without explicitly mentioning SL or the deaf community as a cultural minority, which allowed domestic policy to continue interpreting disability from the logic of rehabilitation.

Consequently, the reception of these norms in Chile was mediated by an assistance-based model. Law N.º 19.284 (1994) ^16^, in force during that period, defined people with disabilities from a perspective of individual lack and deficit, without questioning the system's structural barriers. This enabled the health sector to conceptualize deafness as a biomedical condition, reducing the Deaf experience to the lack of hearing. On this basis, institutional audism was consolidated in MINSAL, understood as a systematic practice that hierarchizes hearing and oralism over the visual-gestural identity of deaf people.

Examination of the GES (Explicit Health Guarantees) clinical guidelines for hearing loss in premature infants (2005) ^18^, hearing loss in individuals over 65 years old (2007) ^19^, and cochlear implants (2008) ^20^, shows the consolidation of a medical-rehabilitative approach. These documents focused public resources on *screening* and prosthetic intervention, systematically excluding sign language (SL) from the therapeutic itinerary. Thus, protected by the general disability regulations, the health system reduced the deaf subject to a «body to be rehabilitated», ignoring their social dimension.

The paradigmatic tension erupted with the ratification of the Convention on the Rights of Persons with Disabilities (CRPD) in 2008 ^14^. This binding international instrument obliged a transition from a medical approach to a rights-based one. The Chilean legislative response was the promulgation of Law N.º 20.422 in 2010 ^22^. Although this norm constituted an advance by establishing principles of equality of opportunities and mentioning SL as a means of access to information, its approach was functional and not cultural.

The original text revealed the State's resistance to recognizing the political autonomy of the community: through a transitory article, it subjected the definition of SL to the administrative guardianship of the State (MIDEPLAN/MINEDUC), instead of recognizing it as a pre-existing cultural heritage. This limitation allowed the health sector to interpret the norm as a primarily accessibility obligation, without substantive changes at the cultural level. Law N.º 20.602 (2012) ^23^ repealed that transitory article, but identity recognition remained weak in healthcare practice.

### The Implementation Gap: Power Asymmetry and the Fragmentation of the State Response (2011-2020)

During this decade, the disconnect between the legislative narrative of inclusion and clinical practice became structural. While Congress approved Law N.º 20.927 (2016) ^26^ obliging television to incorporate SL and subtitles, the health system remained impermeable to the cultural perspective. The National Inclusion Policy 2013-2020 ^25^, although it mentioned access to health, omitted specific guidelines for the deaf community. In parallel, the 2013 GES clinical guideline ^24^ perpetuated the traditional rehabilitative approach. Only in 2019, the Clinical Guideline for children under 4 years of age ^27^ marked a timid turning point by suggesting the incorporation of SL, but it did so from an instrumental logic—as support for oral development—and not as the recognition of an inalienable cultural right.

In the absence of a policy that validated deaf culture within hospitals, a marked power asymmetry was established. Civil society (FUNDESOR) had to fulfill the technical role of the State through an intense advocacy agenda under the Lobby Law, holding hearings with different MINSAL entities ^32^^,^^33^^,^^36^. These efforts sought to institutionalize a Health Unit for Deaf People (USS, for its acronym in Spanish), without achieving a systemic response that would scale these solutions to national public policy. Despite the lack of a national policy, independent local milestones were achieved through specific agreements, such as Exempt Resolution Nº 3119 (2017) between the North Metropolitan Health Service and the Municipality of Recoleta ^34^, and the opening of the USS at the Santa Laura Family Health Center (CESFAM) in El Bosque in 2018 ^35^, demonstrating that the solution was viable, but depended on municipal will and not on a central directive.

This fragmentation of the state response generated an evident territorial inequality, where access became dependent on the patient's postal code. In parallel, the legislative branch attempted to pressure the executive branch to correct this through Resolution Project Nº 1178 (2020), requesting the mandatory presence of personnel trained in SL in health facilities, an initiative that evidenced the lack of administrative response from MINSAL ^37^.

### Judicialization and the paradox of legal recognition without health guarantee (2021-2024)

The recent period evidences the coexistence of a strengthened legal framework with insufficient clinical protection in practice. The promulgation of Law N.º 21.303 (2021) ^29^ marked a historic milestone by explicitly recognizing SL as cultural heritage and natural language, obliging the State to definitively transition from the assistance-based model to one of human rights.

However, MINSAL has prioritized technocratic and vertical measures. Instead of integrating Deaf cultural competence into in-person clinical teams, the authority opted for acultural technological solutions: it incorporated general training goals in the National Plan 2021-2030 ^30^ and implemented video-interpretation in «Salud Responde» ^31^.

However, these unilaterally designed measures assume that the communicative barrier is resolved with remote translation, ignoring the complexity of the therapeutic relationship. The foregoing motivated new requests for hearings via the Lobby Law by civil society before the Undersecretariat of Public Health in May and September 2022 ^38^^,^^39^. These union efforts sought structural solutions for hospitals, but did not obtain a systemic response.

Given the failure of the political path to achieve institutional changes, the crisis shifted to the individual clinical reality. Administrative failure left patients unprotected in their most critical moments, forcing individuals —sometimes supported by local associations— to activate legal action not as a political strategy, but as a survival mechanism.

The very existence of these protection resources constitutes factual evidence that neither training nor the remote platform are solving the problem at the point of care. The rulings of the Courts of Appeals of Concepcion and San Miguel ^5^^-^^7^ documented this reality: public hospitals unable to communicate with an infant, a pregnant woman, and an oncology patient. These cases demonstrate that, in real emergencies, technological tools do not replace the cultural mediator and that individuals must bear the burden of litigating to access a basic right. However, in May 2024, the Supreme Court revoked this last ruling ^8^.

This final ruling evidences the structural disconnection: the system validates the dependence on external municipal resources or third-party initiatives, instead of demanding its own installed capacity in high-complexity hospitals. This judicial setback, added to FUNDESOR's insistence on new hearings during 2022 ^38^^,^^39^, shows that, despite legislative protection, the Chilean health system still does not guarantee the linguistic autonomy of deaf patients as an inherent institutional service, depending on the goodwill of local initiatives or the outsourcing of services.

## DISCUSSION

The analysis of the Chilean regulatory trajectory suggests that the exclusion of the deaf community in health is not due to a legal vacuum, but to an ideological resistance of a structural nature. While the Legislative Power has progressively transitioned towards the social model—aligning with the International Convention ^14^ and recognizing SL as cultural heritage in Law N.º 21.303 ^(^^29^—the healthcare system maintains a «clinical inertia» anchored to the medical-rehabilitative paradigm. Recent evidence on the parliamentary discussion of this law confirms that the legislator explicitly sought to differentiate the «deaf person» as a subject of a cultural minority, distancing them from the clinical category of «hearing disability» ^40^. However, this political distinction has been ignored by the health authority. This disconnection explains why, despite explicit inclusion mandates, care practices remain discriminatory and dependent on judicialization ^4^.

The hegemony of institutional audism: MINSAL's resistance configures what the literature defines as «institutional audism» ^2^^,^^3^: a governance structure that only validates hearing and orality as health norms. The persistence of GES clinical guidelines that for two decades exclusively prioritized prosthetic intervention ^18^^,^^19^^,^^20^, invisibilizing deaf linguistic identity, demonstrates that the health authority conceives the deaf patient as a «biological body to be repaired» and not as a subject of cultural rights. This biomedical vision has prevented LS from being considered a legitimate health service, relegating it to a secondary role compared to auditory technology, as observed even in the most recent guidelines that subordinate it to auditory-verbal therapy ^27^.

Technocratic verticality versus social demand: The study evidences a marked power asymmetry in decision-making. Unlike other groups that co-construct policies with MINSAL, Deaf civil society has been kept on the periphery. The analyzed lobby records demonstrate that organizations such as FUNDESOR attempted for years to propose face-to-face structural solutions (specialized health units) to the Primary Health Care Division and the Undersecretary of Public Health ^32^^,^^33^^,^^36^, only to encounter administrative inaction. This dynamic of exclusion is not exclusive to the health sector; recent research shows that, even during the processing of Law N.º 21.303, the technical commissions introduced critical modifications —such as the authorization for hearing people to teach SL— without consulting the deaf community, replicating a hegemonic logic that silences the expert voice of the community itself ^40^.

Given this demand for cultural relevance, the State responded with technocratic verticality. The implementation of «Salud Responde» with video-interpretation ^31^ and general training goals in the National Auditory Health Plan ^30^ reflects an attempt to solve a complex problem of therapeutic bond through logistical remote translation tools. This operational logic is consistent with the findings of the legislative analysis of Law N.º 21.303, where it was identified that part of the political class conceives SL as a communication tool «replaceable» or substitutable by writing and technological means, disregarding its status as an irreplaceable natural language for the construction of meaning ^40^. This response, designed without binding community validation, erroneously assumes that the barrier is merely idiomatic and not cultural. The existence of protection resources due to lack of critical care in situations of childbirth and oncology ^5^^-^^7^ constitutes factual evidence of the failure of this approach: remote technology failed to replace the cultural competence necessary at the in-person point of care.

The institutionalization of precariousness: The risk of outsourcing. A critical finding of this analysis is the system's tendency to outsource the guarantee of rights. The recent Supreme Court ruling ^8^, which validated that Hospital Barros Luco relied on a municipal clinic (USS El Bosque) to interpret a patient, sets a precedent for inequity. By legitimizing that a high-complexity hospital depends on the installed capacity of an external municipality ^35^ or on temporary local agreements ^34^, the State renounces its duty to guarantee universal accessibility in its central network. This transforms the right to health into a territorial lottery, where dignified care depends on the political and budgetary will of local governments and not on a guaranteed national policy.

## LESSONS AND RECOMMENDATIONS FOR PUBLIC POLICY

To overcome this implementation gap and transition from «deficit care» to «intercultural health», three structural measures are proposed: 

1. Institutionalization of linguistic mediation: MINSAL must overcome voluntarism and standardize the experience of the Special Program for Health and Indigenous Peoples (PESPI). It is necessary to create structural, centrally funded positions for Deaf intercultural facilitators and interpreters across the hospital network to guarantee face-to-face reception and informed consent.

2. Reformulation of clinical guidelines with a rights-based approach: It is imperative to update the hearing loss GES decrees ^18^^,^^24^^,^^27^ so that the teaching of SL and the accompaniment of Deaf identity become guaranteed and funded services at the same level as auditory-verbal therapies, eliminating the bias that presents them as mutually exclusive options.

3. Scaling of the Reference Units model: The USS of El Bosque ^35^ demonstrates that primary health care is the ideal space for cultural appropriateness. The State must stop relying on municipal pilots and formalize a national policy of reference units in each health service to act as a resolutive gateway.

## CONCLUSIONS

The analyzed evidence reveals that, after decades of normative evolution, the exclusion of the deaf community in the Chilean health system persists without a structural solution. This problem is not a consequence of a legal vacuum, but rather of a political and ideological resistance from the health apparatus. Although the State has modernized its legislation aligning with human rights standards, MINSAL has maintained an audist governance that instrumentalizes SL as an accessory technical tool, denying its status as a fundamental cultural right.

This study highlights that the state response to the demand for linguistic autonomy has been technocratic and vertical. The insistence on implementing remote translation solutions without community validation, coupled with the judicial validation of the outsourcing of services to municipal primary care, has institutionalized territorial inequity that precariousizes high-complexity care.

To settle this historical debt, it is not enough to appeal to «political will» nor to enact new laws. It is necessary to reorient the care model from a rights-based approach that ensures linguistic access as an enforceable guarantee and recognizes the deaf community as a collective subject, with effective participation in healthcare decisions. This implies overcoming rehabilitative paternalism and institutionalizing intercultural facilitators and specialized reference units as permanent components of the hospital network. In parallel, it is key to strengthen staff training and public education to denaturalize audism and consolidate the social recognition of SL and deaf culture. As a projection, future research should evaluate the implementation of these devices in the public health system and their effects on continuity of care, clinical safety, and territorial equity.
