# Supplementary material for: Gaps between normative advancement and clinical response: the inclusion of the deaf community in chilean public health
Source: Rev Peru Med Exp Salud Publica. 2026 Jun 25;43(2):251–9. doi: 10.17843/rpmesp.2026.432.15099 (PMC13456920; doi:10.17843/rpmesp.2026.432.15099)
Supplement: Translation

# Translation: Brechas entre el avance normativo y la respuesta clínica: la inclusión de la comunidad sorda en la salud pública chilena

**Authors:** Valeria Campos, Irene Villalobos-Saldivia, Sebastián Quiñones

**Affiliations:** 1 Departamento de Salud Pública, Facultad de Medicina, Universidad San Sebastián, Sede Tres Pascualas, Octava, Chile.; 2 Facultad de Ciencias Sociales, Escuela de Psicología, Universidad Santo Tomás, Santiago, Chile.; 3 Universidad de Santiago de Chile, Santiago de Chile; Chile.

**Keywords:** Sordera, Personas con Discapacidad, Sistemas de Salud, Legislación Sanitaria, Derechos Humanos, Lengua de Señas

## Abstract

Chile ha experimentado una evolución progresiva de su marco normativo hacia un enfoque de derechos; sin embargo, la exclusión de la comunidad Sorda en el sistema de salud persiste. Esta investigación analiza las brechas político-institucionales entre el marco normativo de derechos y las prácticas clínicas del sistema de salud chileno. Los hallazgos evidencian un desacople estructural vinculado al audismo institucional, que reduce la Lengua de Señas a un recurso instrumental y prioriza soluciones tecnocráticas de interpretación remota por sobre prácticas presenciales de competencia cultural y comunicativa. Además, la judicialización ha contribuido a legitimar la externalización de garantías hacia el nivel municipal, profundizando inequidades territoriales y precarizando el acceso, especialmente en la atención de complejidad. Se concluye que los derechos lingüísticos no pueden depender de voluntades administrativas y requieren una reorientación político-institucional con incorporación estructural de facilitadores interculturales en la red sanitaria.

## INTRODUCCIÓN

Históricamente, la comprensión de la sordera ha transitado desde una visión patológica hacia un enfoque de derechos humanos. Bajo el paradigma médico-rehabilitador tradicional, la persona sorda era considerada un «cuerpo con déficit» que requería corrección para integrarse a la norma oyente. Esta perspectiva, denominada audismo, opera como una estructura de discriminación que valora la capacidad de oír y hablar sobre la experiencia visual y gestual, ignorando la dimensión psicosocial de la sordera ^1^^-^^3^. Sin embargo, la emergencia del modelo social de la discapacidad y los estudios sordos (*Deaf Studies)* han redefinido esta condición no como una carencia, sino como una experiencia de diversidad humana que da lugar a una comunidad con una identidad, historia y lengua propia a través de la lengua de señas (LS) ^2^^,^^3^.

Desde la salud pública, este cambio de paradigma es decisivo porque sitúa a la comunidad sorda como una minoría lingüística cuyo acceso depende de la adecuación de los entornos sanitarios. El enfoque sociocultural y los estudios sordos han consolidado esta mirada al reconocer una comunidad con identidad y cultura propias, sustentada en el uso de la LS, en contraste con el modelo médico ^2^^,^^3^. En articulación con el discurso de la discapacidad, ello ha favorecido el reconocimiento de derechos y la exigencia de ajustes para garantizar acceso equitativo a salud, educación y otros servicios ^2^^,^^3^. Sin embargo, a nivel global, cerca de 70 millones de personas sordas continúan enfrentando barreras en salud, principalmente por dificultades de comunicación ^4^. La evidencia muestra que esta brecha opera como determinante social de la salud, incrementando el riesgo de diagnósticos erróneos, debilitando la adherencia terapéutica, vulnerando el consentimiento informado y asociándose a peores resultados sanitarios ^4^. Por tanto, el acceso lingüístico constituye un imperativo ético y de derechos humanos.

En Chile se estima que viven más de 500 mil personas sordas o con discapacidad auditiva, quienes continúan enfrentando limitaciones de acceso y formas persistentes de exclusión ante la insuficiencia de políticas estatales que incorporen de manera efectiva el enfoque sociocultural ^4^. Si bien los avances normativos y la ratificación de tratados internacionales han establecido obligaciones explícitas de resguardo de sus derechos lingüísticos y culturales, incluyendo la autonomía lingüística reconocida en leyes recientes como la Ley N.º 21.303 ^4^, estos marcos no se han traducido de forma consistente en transformaciones sostenidas dentro de la institucionalidad de la salud pública. En este contexto, la creciente judicialización de la atención en salud expresa la brecha entre el reconocimiento legal y la práctica sanitaria, y sugiere la persistencia de dinámicas audistas que dificultan la incorporación efectiva de la LS y perpetúan la exclusión ^5^^-^^8^.

Ante esta desconexión persistente, resulta urgente examinar las barreras estructurales que dificultan la traducción de los mandatos legales en prácticas y dispositivos efectivos dentro del sistema de salud. En ese sentido, este estudio analiza las brechas político-institucionales entre el marco normativo de derechos y las prácticas clínicas en Chile, identificando los factores históricos y actuales que restringen el reconocimiento de la comunidad Sorda como sujeto de derechos en salud.

## ABORDAJE METODOLÓGICO

El estudio adoptó un enfoque cualitativo ^9^ y se desarrolló entre 2024 y 2025. Se realizó un análisis documental normativo ^10^, entendido como una revisión sistemática y comparada de textos legales y técnicos orientada a extraer mandatos, definiciones, estándares y procedimientos exigibles del marco regulatorio del sector salud en Chile. El corpus incluyó leyes, decretos, reglamentos, normas técnicas, protocolos, orientaciones ministeriales y tratados internacionales ratificados. 

La búsqueda abarcó literatura científica y gris publicada entre 1950 y 2024. Se consultaron bases de datos indexadas (PubMed, Web of Science, SciELO y Google Scholar) y repositorios de organismos internacionales (Organización de Naciones Unidas [ONU], Organización Mundial de la Salud [OMS], Organización de los Estados Americanos [OEA]) y nacionales (Biblioteca del Congreso Nacional, Ministerio de Salud de Chile [MINSAL], Servicio Nacional de la Discapacidad [SENADIS]). La estrategia combinó descriptores DeCS/MeSH y términos libres en español e inglés, incorporando también denominaciones históricas para reconstruir la evolución normativa. 

El objetivo de la investigación fue analizar las brechas político-institucionales entre el marco normativo de derechos y las prácticas clínicas del sistema de salud chileno, identificando barreras históricas y vigentes que limitan el reconocimiento de la comunidad Sorda como sujeto de derechos en salud. Con el fin de operacionalizar este propósito y orientar el análisis, se definieron dos objetivos específicos: identificar y caracterizar las temáticas, enfoques y definiciones presentes en los marcos normativos de salud referidos a la comunidad Sorda, considerando su evolución histórica.

El proceso de selección y análisis documental se organizó en seis fases, con participación del equipo investigador en cada etapa mediante trabajo conjunto, deliberación y control de la codificación y la síntesis, buscando fortalecer la consistencia analítica y el rigor metodológico ^10^. Las fases incluyeron: (a) definición del propósito y alcance; (b) establecimiento de criterios de inclusión y exclusión; (c) diseño y ejecución de la búsqueda; (d) conformación, registro y organización del corpus; (e) categorización temática; y (f) síntesis e integración de resultados. En todo el procedimiento se resguardaron directrices éticas propias de la investigación cualitativa ^10^.

Posteriormente, los documentos se ordenaron cronológicamente y se codificaron en ATLAS.ti; esta estrategia permitió sistematizar el corpus, reconstruir su evolución histórica y establecer categorías temáticas con sus respectivos códigos analíticos. El corpus final incluyó 26 documentos del periodo 1982-2023: cinco instrumentos internacionales oficiales (tabla 1), principalmente de la ONU, y documentos nacionales (tabla 2 y 3) correspondientes a leyes, guías clínicas, políticas públicas, fallos judiciales, audiencias, decretos, un proyecto de resolución y un convenio. Para la visualización de la síntesis de resultados (figura 1). Además, se utilizaron herramientas de inteligencia artificial generativa con fines exclusivamente de diseño gráfico, utilizando la base de datos estructurada y validada por el equipo investigador. En conjunto, el procedimiento consolidó un corpus documental sistematizado, trazable y apto para la interpretación normativa.

**Tabla 1:** Evolución de instrumentos internacionales sobre discapacidad y derechos de la comunidad sorda (1982-2017).

Título	Organismo	Año	Propósito y contenidos Clave	Carácter jurídico
Programa de Acción Mundial para las Personas con Discapacidad ^11^	ONU	1982	Promover medidas eficaces para la prevención de la incapacidad, la rehabilitación y la realización de los objetivos de «participación plena» de las personas con discapacidad en la vida social y el desarrollo y de «igualdad».	No vinculante
Normas Uniformes sobre la Igualdad de Oportunidades para las Personas con Discapacidad ^12^	ONU	1993	Garantizar que niñas, niños, mujeres y hombres con discapacidad, en su calidad de miembros de sus respectivas sociedades, puedan ejercer los mismos derechos y obligaciones que los demás. Define mecanismos de supervisión estatal.	No vinculante
Convención Interamericana para la Eliminación de todas las Formas de Discriminación contra las Personas con Discapacidad ^13^	OEA	1999	Prevenir y eliminar todas las formas de discriminación contra las personas con discapacidad y propiciar su plena integración en la sociedad. Aporta una regulación más amplia que los instrumentos generales previos.	Vinculante
Convención de los Derechos de las Personas con Discapacidad ^14^	ONU	2008	Promover, proteger y asegurar el goce pleno y en condiciones de igualdad de todos los derechos humanos y libertades fundamentales. Marca el cambio de paradigma al reconocer explícitamente la identidad cultural y lingüística de las personas sordas.	Vinculante
Día Internacional de las Lenguas de Señas ^15^	ONU	2017	Concienciar sobre la importancia de las lenguas de señas para la plena realización de los derechos humanos de las personas sordas. Resolución A/72/439.	No vinculante

ONU: Organización Naciones Unidas, OEA: Organización de los Estados Americanos.

**Tabla 2:** Trayectoria normativa y técnica: leyes, guías clínicas y políticas públicas de salud (1994-2024).

Año	Documento/título	Institución	Propósito y contenido	Tipo de Instrumento
1994	Ley N.º 19.284 ^16^	Ministerio de Planificación y Cooperación	Establecer la forma y condiciones que permitan obtener la plena integración de las personas con discapacidad en la sociedad, y velar por el pleno ejercicio de los derechos que la Constitución y las leyes reconocen.	Ley
2002	Decreto 99 ^17^	Ministerio de Relaciones Exteriores	Aprobar y promulgar la Convención Interamericana para la Eliminación de Todas las Formas de Discriminación contra las Personas con Discapacidad en Chile.	Decreto
2005	Guía clínica GES: Hipoacusia neurosensorial del prematuro ^18^	Ministerio de Salud, Fondo Nacional de Salud, Junta Nacional de Auxilio Escolar y Becas y Fondo Nacional de la Discapacidad	Crear un programa nacional de detección temprana de hipoacusia en prematuros de muy bajo peso, estableciendo pautas para el screening, diagnóstico, tratamiento y rehabilitación.	Guía clínica
2007	Guía clínica: Hipoacusia en personas de 65 años y más ^19^	Ministerio de Salud	Mejorar la calidad de vida y reducir la morbimortalidad en adultos mayores con hipoacusia bilateral, optimizar la prescripción y adaptación de audífonos y aumentar la adherencia a su uso.	Guía clínica
2008	Guía clínica: implante Coclear ^20^	Ministerio de Salud	Proporcionar recomendaciones para la rehabilitación de candidatos a implante coclear, optimizando el proceso en la red de salud y facilitando la toma de decisiones del equipo profesional.	Guía clínica
2008	Decreto 201 ^21^	Ministerio de Relaciones Exteriores	Aprobar y promulgar la Convención de los Derechos de las Personas con Discapacidad (CDPD) y su Protocolo Facultativo.	Decreto
2010	Ley N.º 20.422 ^22^	Ministerio de Planificación	Garantizar la igualdad de oportunidades y la inclusión social, asegurando el pleno disfrute de derechos y eliminando la discriminación.	Ley
2012	Ley N.º 20.602 ^23^	Ministerio de Desarrollo Social; Subsecretaría de Servicios Sociales	Derogar el inciso sexto del artículo primero transitorio de la Ley 20.422, que establecía que «El Estado, en conjunto con la comunidad... definirá, en un plazo de tres años, el lenguaje de señas chileno».	Ley
2013	Guía clínica AUGE: Hipoacusia en menores de 2 años ^24^	Ministerio de Salud	Brindar un enfoque integral para la detección temprana, diagnóstico y tratamiento. Incluye uso de audífonos o implantes y terapia del lenguaje para mejorar el desarrollo comunicativo.	Guía clínica
2013	Política Nacional para la Inclusión Social 2013-2020 ^25^	Servicio Nacional de la Discapacidad, Ministerio de Desarrollo Social	Fomentar el respeto de derechos y generar acceso a servicios de salud inclusivos y especializados, centrándose en prevención, tratamiento, habilitación y rehabilitación.	Política pública
2016	Ley N.º 20.927 ^26^	Ministerio de Desarrollo Social	Asegurar el acceso a la información en TV, obligando a los canales a usar subtítulos y lengua de señas en programas importantes como noticias de emergencia y campañas públicas.	Ley
2019	Guía clínica: Hipoacusia en menores de 4 años ^27^	Ministerio de Salud	Recomendaciones para detección y tratamiento, enfocándose en optimizar dispositivos de ayuda auditiva y terapia del lenguaje para asegurar inclusión social.	Guía clínica
2020	Programa Salud Responde (Implementación) ^28^	Ministerio de Salud	Incorporación de orientación accesible en situaciones de urgencia y consultas administrativas a través de canales inclusivos.	Política pública
2021	Ley N.º 21.303 ^29^	Ministerio de Desarrollo Social y Familia	Promover el uso de la lengua de señas chilena, asegurando el acceso a la información y servicios. Reconoce a la LS como patrimonio cultural y lengua natural.	Ley
2022	Plan Nacional de Salud Auditiva 2021-2030 ^30^	Ministerio de Salud	Mejorar la salud auditiva y prevenir la pérdida a través del diagnóstico temprano, tratamiento y rehabilitación, promoviendo la inclusión social.	Política pública
2024	Programa Salud Responde (Video-interpretación) ^31^	Ministerio de Salud	Incorporación de Lengua de Señas Chilena para la atención en salud de manera remota mediante video.	Política pública

**Tabla 3:** Movilización social y judicialización: brecha de implementación y respuesta ciudadana (2017-2024).

Año	Hito/acción	Actor institucional involucrado	Descripción del conflicto/demanda
2017	Audiencia de Lobby ^32^	MINSAL (DIVAP)	Fundación Sordos Chilenos solicita a Soledad Turra financiamiento para USS.
2017	Audiencia de Lobby ^33^	Subsecretaría Salud Pública	Fundación Sordos Chilenos solicita a Bernardo Martorell financiamiento para USS.
2017	Resolución Exenta Nº 3119 ^34^	SS Metropolitano Norte / Muni. Recoleta	Convenio local para proyecto de USS.
2018	Apertura USS El Bosque ^35^	Muni. El Bosque / CESFAM Santa Laura	Apertura de la primera USS, gestión municipal sin financiamiento estructural central.
2019	Audiencia de Lobby ^36^	MINSAL (DIVAP)	Fundación Sordos, Federación Nacional y CESFAM Santa Laura insisten en financiamiento USS.
2022	Proyecto de Resolución Nº 1178 ^37^	Cámara de Diputados	El Legislativo solicita al Ejecutivo obligatoriedad de personal capacitado en LS en salud pública.
2022	Audiencia de Lobby ^38^	Subsecretaría Salud Pública	Fundación Sordos y Federación de Intérpretes solicitan al Subsecretario Cuadrado financiamiento USS.
2022	Audiencia de Lobby ^39^	Redes Asistenciales / DIVAP	Solicitud de mesa de trabajo conjunta para abordar acceso a salud mediante propuesta comunitaria.
2023	Fallo Rol 66.839-2022 ^5^	Corte de Apelaciones Concepción	Ordena intérprete permanente en Hospital Grant Benavente (caso lactante).
2023	Fallo Rol 100.326-2022 ^6^	Corte de Apelaciones Concepción	Acoge recurso contra CESFAM Víctor M. Fernández por desatención a gestante sorda.
2024	Fallo Rol 4070-2023 ^7^	Corte de Apelaciones San Miguel	Ordena a Hospital Barros Luco garantizar intérprete para paciente oncológica.
2024	Revocación Rol 9.186-2024 ^8^	Corte Suprema	Revoca fallo anterior; valida coordinación externa con CESFAM Santa Laura (municipal) para interpretar.

MINSAL: Ministerio de Salud, USS: Unidad de salud para Personas Sordas, DIVAP: División de Atención Primaria, CESFAM: Centro de Salud Familiar.

## RESULTADOS

El análisis documental evidencia una transición lenta y conflictiva de paradigmas en discapacidad, caracterizada por una brecha estructural entre el avance normativo y la respuesta clínica que se resume cronológicamente en la figura 1.

**Figura 1:** Línea de tiempo comparativa entre la evolución normativa de derechos y la respuesta clínica del sistema de salud chileno (1990-2024).

Fuente: Elaboración propia basada en el análisis documental. El diseño gráfico de esta figura fue asistido por herramientas de inteligencia artificial generativa (NotebookLM/Gemini), bajo supervisión y validación de contenidos por parte de los autores.

### Hegemonía del modelo biomédico y el inicio de la tensión normativa (1982-2010)

A nivel internacional, si bien instrumentos fundacionales como el Programa de Acción Mundial ^11^ y las Normas Uniformes de la ONU (12) incorporaron conceptos generales de igualdad, no reconocían de manera explícita a la cultura sorda y mantenían una noción indiferenciada de la discapacidad. Además, carecían de fuerza vinculante.

En el ámbito regional, Chile dio un paso jurídico importante al ratificar en 2002 el Decreto 99 ^17^, que promulgaba la Convención Interamericana (CIADIS) ^13^. Aunque este fue el primer instrumento vinculante, su texto se centraba en la prevención y la eliminación de la discriminación, sin mencionar explícitamente a la LS ni a la comunidad sorda como minoría cultural, lo que permitió que la política interna siguiera interpretando la discapacidad desde la lógica de la rehabilitación.

En consecuencia, la recepción de estas normas en Chile quedó mediada por un modelo asistencialista. La Ley N.º 19.284 (1994) ^16^, vigente en ese periodo, definió a la persona con discapacidad desde la carencia y el déficit individual, sin cuestionar las barreras estructurales del sistema. Ello habilitó al sector salud a conceptualizar la sordera como condición biomédica, reduciendo la experiencia Sorda a la falta de audición. Sobre esta base se consolidó el audismo institucional en el MINSAL, entendido como una práctica sistemática que jerarquiza la audición y la oralidad por sobre la identidad visual-gestual de las personas sordas.

Al examinar las guías clínicas GES (Garantías Explícitas en Salud) para hipoacusia del prematuro (2005) ^18^, hipoacusia en mayores de 65 años (2007) ^19^ e implante coclear (2008) ^20^, muestra la consolidación de un enfoque médico-rehabilitador. Estos documentos centraron los recursos públicos en el *screening* y la intervención protésica, excluyendo sistemáticamente a la LS del itinerario terapéutico. Así, amparado en la normativa general de discapacidad, el sistema sanitario redujo al sujeto sordo a un «cuerpo a rehabilitar», ignorando su dimensión social.

La tensión paradigmática estalló con la ratificación de la Convención de los Derechos de las Personas con Discapacidad (CDPD) en 2008 ^14^. Este instrumento internacional vinculante obligaba a transitar desde un enfoque médico a uno de derechos. La respuesta legislativa chilena fue la promulgación de la Ley N.º 20.422 en 2010 ^22^. Si bien esta norma constituyó un avance al establecer principios de igualdad de oportunidades y mencionar la LS como medio de acceso a la información, su enfoque fue funcional y no cultural.

El texto original reveló la resistencia del Estado a reconocer la autonomía política de la comunidad: mediante un artículo transitorio, sometió la definición de la LS a la tutela administrativa del Estado (MIDEPLAN/MINEDUC), en lugar de reconocerla como un patrimonio cultural preexistente. Esta limitación permitió que el sector salud interpretara la norma como una obligación principalmente de accesibilidad, sin cambios sustantivos en el plano cultural. La Ley N.º 20.602 (2012) ^23^ derogó ese artículo transitorio, pero el reconocimiento identitario siguió siendo débil en la práctica sanitaria.

### La brecha de implementación: asimetría de poder y la fragmentación de la respuesta estatal (2011-2020)

Durante esta década, la desconexión entre la narrativa legislativa de inclusión y la práctica clínica se hizo estructural. Mientras el Congreso aprobaba la Ley N.º 20.927 (2016) ^26^ obligando a la televisión a incorporar LS y subtítulos, el sistema de salud se mantuvo impermeable a la perspectiva cultural. La Política Nacional de Inclusión 2013-2020 ^25^, si bien mencionaba el acceso a salud, omitió lineamientos específicos para la comunidad sorda. En paralelo, la guía clínica GES de 2013 ^24^ perpetuó el enfoque rehabilitador tradicional. Recién en 2019, la Guía Clínica para menores de 4 años ^27^ marcó un tímido punto de inflexión al sugerir la incorporación de la LS, pero lo hizo desde una lógica instrumental -como apoyo al desarrollo oral- y no como el reconocimiento de un derecho cultural inalienable.

Ante la ausencia de una política que validara la cultura sorda dentro de los hospitales, se configuró una marcada asimetría de poder. La sociedad civil (FUNDESOR) debió suplir el rol técnico del Estado mediante una intensa agenda de incidencia bajo la Ley de Lobby, sosteniendo audiencias con distintas entidades del MINSAL ^32^^,^^33^^,^^36^. Estas gestiones buscaban institucionalizar una Unidad de salud para Personas Sordas (USS), sin lograr una respuesta sistémica que escalara estas soluciones a política pública nacional. Pese a la falta de una política nacional, se lograron hitos locales independientes mediante convenios específicos, como la Resolución Exenta Nº 3119 (2017) entre el Servicio de Salud Metropolitano Norte y la Municipalidad de Recoleta ^34^, y la apertura de la USS en el Centro de Salud Familiar (CESFAM) Santa Laura de El Bosque en 2018 ^35^, demostrando que la solución era viable, pero dependía de la voluntad municipal y no de una directriz central.

Esta fragmentación de la respuesta estatal generó una inequidad territorial evidente, donde el acceso pasó a depender del código postal del paciente. En paralelo, el poder legislativo intentó presionar al ejecutivo para corregir esto mediante el Proyecto de Resolución Nº 1178 (2020), solicitando la obligatoriedad de personal capacitado en LS en recintos de salud, iniciativa que evidenció la falta de respuesta administrativa del MINSAL ^37^. 

### Judicialización y la paradoja del reconocimiento legal sin garantía sanitaria (2021-2024)

El periodo reciente evidencia la coexistencia de un marco legal fortalecido con una protección clínica insuficiente en la práctica. La promulgación de la Ley N.º 21.303 (2021) ^29^ marcó un hito histórico al reconocer explícitamente a la LS como patrimonio cultural y lengua natural, obligando al Estado a transitar definitivamente desde el modelo asistencialista hacia uno de derechos humanos.

Sin embargo, el MINSAL ha privilegiado medidas tecnocráticas y verticales. En lugar de integrar la competencia cultural sorda en los equipos clínicos presenciales, la autoridad optó por las soluciones tecnológicas aculturales: incorporó metas de capacitación generales en el Plan Nacional 2021-2030 ^30^ e implementó la video-interpretación en «Salud Responde» ^31^.

No obstante, estas medidas diseñadas unilateralmente, asumen que la barrera comunicativa se resuelve con traducción remota, ignorando la complejidad del vínculo terapéutico. Lo anterior motivó nuevas solicitudes de audiencias vía Ley de lobby por parte de la sociedad civil ante la Subsecretaría de Salud Pública en mayo y septiembre de 2022 ^38^^,^^39^. Estas gestiones gremiales buscaban soluciones estructurales para los hospitales, pero no obtuvieron una respuesta sistémica.

Ante el fracaso de la vía política para lograr cambios institucionales, la crisis se trasladó a la realidad clínica individual. La insuficiencia administrativa dejó desprotegidos a los pacientes en sus momentos más críticos, forzando a personas naturales -en ocasiones respaldadas por asociaciones locales- a activar la vía judicial no como estrategia política, sino como mecanismo de supervivencia.

La propia existencia de estos recursos de protección constituye la evidencia fáctica de que ni las capacitaciones ni la plataforma remota están resolviendo el problema en el punto de atención. Los fallos de las cortes de apelaciones de Concepción y San Miguel ^5^^-^^7^ documentaron esta realidad: hospitales públicos incapaces de comunicarse con un lactante, una gestante y una paciente oncológica. Estos casos demuestran que, en la urgencia real, las herramientas tecnológicas no reemplazan al mediador cultural y que son los individuos quienes deben cargar con el peso de litigar para acceder a un derecho básico. Sin embargo, en mayo de 2024, la Corte Suprema revocó este último fallo ^8^.

Este dictamen final evidencia la desconexión estructural: el sistema valida la dependencia de recursos municipales externos o iniciativas de terceros, en lugar de exigir capacidad instalada propia en los hospitales de alta complejidad. Este revés judicial, sumado a la insistencia de FUNDESOR en nuevas audiencias durante 2022 ^38^^,^^39^, pone de manifiesto que, pese al blindaje legislativo, el sistema de salud chileno aún no garantiza la autonomía lingüística del paciente sordo como una prestación institucional propia, dependiendo de la buena voluntad de iniciativas locales o de la externalización de servicios.

## DISCUSIÓN

El análisis de la trayectoria normativa chilena sugiere que la exclusión de la comunidad sorda en salud no obedece a un vacío legal, sino a una resistencia ideológica de carácter estructural. Mientras el Poder Legislativo ha transitado progresivamente hacia el modelo social -alineándose con la Convención Internacional ^14^ y reconociendo a la LS como patrimonio cultural en la Ley N.º 21.303 ^29^-, el sistema sanitario mantiene una «inercia clínica» anclada al paradigma médico-rehabilitador. Evidencia reciente sobre la discusión parlamentaria de esta ley confirma que el legislador buscó explícitamente diferenciar a la «persona sorda» como sujeto de una minoría cultural, distanciándola de la categoría clínica de « discapacidad auditiva» ^40^. Sin embargo, esta distinción política ha sido ignorada por la autoridad sanitaria. Esta desconexión explica por qué, pese a existir mandatos de inclusión explícitos, las prácticas de atención siguen siendo discriminatorias y dependientes de la judicialización ^4^.

La resistencia del MINSAL configura lo que la literatura define como «audismo institucional» ^2^^,^^3^: una estructura de gobernanza que valida únicamente la audición y la oralidad como normas de salud. La persistencia de guías clínicas GES que durante dos décadas priorizaron exclusivamente la intervención protésica ^18^^,^^19^^,^^20^, invisibilizando la identidad lingüística sorda, demuestra que la autoridad sanitaria concibe al paciente sordo como un «cuerpo biológico a reparar» y no como un sujeto de derechos culturales. Esta visión biomédica ha impedido que la LS sea considerada una prestación de salud legítima, relegándola a un rol secundario frente a la tecnología auditiva, tal como se observa incluso en las guías más recientes que la subordinan a la terapia auditivo-verbal ^27^.

El estudio evidencia una marcada asimetría de poder en la toma de decisiones. A diferencia de otros gremios que co-construyen políticas con el MINSAL, la sociedad civil sorda ha sido mantenida en la periferia. Los registros de lobby analizados demuestran que organizaciones como FUNDESOR intentaron durante años proponer soluciones estructurales presenciales (unidades de salud especializadas) ante la División de Atención Primaria y la Subsecretaría de Salud Pública ^32^^,^^33^^,^^36^, encontrándose con la inacción administrativa. Esta dinámica de exclusión no es exclusiva del sector salud; investigaciones recientes demuestran que, incluso durante la tramitación de la Ley N.º 21.303, las comisiones técnicas introdujeron modificaciones críticas -como la autorización para que oyentes enseñen LS- sin consultar a la comunidad sorda, replicando una lógica hegemónica que silencia la voz experta de la propia comunidad ^40^.

Ante esta demanda de pertinencia cultural, el Estado respondió con verticalidad tecnocrática. La implementación de «Salud Responde» con video-interpretación ^31^ y metas de capacitación generales en el Plan Nacional de Salud Auditiva ^30^ refleja un intento de resolver un problema complejo de vínculo terapéutico mediante herramientas logísticas de traducción remota. Esta lógica operativa es coherente con los hallazgos del análisis legislativo de la Ley N.º 21.303, donde se identificó que parte de la clase política concibe a la LS como una herramienta de comunicación «reemplazable» o sustituible por la escritura y medios tecnológicos, desestimando su estado de lengua natural irreemplazable para la construcción de sentido ^40^. Esta respuesta, diseñada sin validación comunitaria vinculante, asume erróneamente que la barrera es meramente idiomática y no cultural. La existencia de recursos de protección por falta de atención crítica en situaciones de parto y oncología ^5^^-^^7^ constituye la evidencia fáctica del fracaso de este enfoque: la tecnología remota no logró reemplazar la competencia cultural necesaria en el punto de atención presencial.

Un hallazgo crítico de este análisis es la tendencia del sistema a externalizar la garantía de derechos. El reciente fallo de la Corte Suprema ^8^, que validó que el Hospital Barros Luco se apoyara en un consultorio municipal (USS El Bosque) para interpretar a una paciente, sienta un precedente de inequidad. Al legitimar que un hospital de alta complejidad dependa de la capacidad instalada de un municipio externo ^35^ o de convenios locales transitorios ^34^, el Estado renuncia a su deber de garantizar accesibilidad universal en su red central. Esto transforma el derecho a la salud en una lotería territorial, donde la atención digna depende de la voluntad política y presupuestaria de los gobiernos locales y no de una política nacional garantizada.

## LECCIONES Y RECOMENDACIONES DE POLÍTICA PÚBLICA

Para superar esta brecha de implementación y transitar desde la «atención del déficit» a la «salud intercultural», se proponen tres medidas estructurales:

1. Institucionalización de la mediación lingüística: el MINSAL debe superar el voluntarismo y homologar la experiencia del Programa Especial de Salud y Pueblos Indígenas (PESPI). Se requiere crear cargos estructurales de facilitadores interculturales sordos e intérpretes en la red hospitalaria, financiados centralmente, para garantizar la acogida y el consentimiento informado presencial.

2. Reformulación de las guías clínicas con enfoque de derechos: es imperativo actualizar los decretos GES de hipoacusia ^18^^,^^24^^,^^27^ para que la enseñanza de la LS y el acompañamiento a la identidad sorda sean prestaciones garantizadas y financiadas al mismo nivel que las terapias auditivo-verbales, eliminando el sesgo que las presenta como opciones excluyentes.

3. Escalamiento del modelo de Unidades de Referencia: la experiencia de la USS de El Bosque ^35^ demuestra que la atención primaria es el espacio idóneo para la pertinencia cultural. El Estado debe dejar de depender de pilotos municipales y formalizar una política nacional de unidades de referencia en cada servicio de salud, que actúen como puerta de entrada resolutiva.

## CONCLUSIONES

La evidencia analizada revela que, tras décadas de evolución normativa, la exclusión de la comunidad sorda en el sistema de salud chileno persiste sin una solución estructural. Este problema no es consecuencia de un vacío legal, sino de una resistencia política e ideológica del aparato sanitario. Si bien el Estado ha modernizado su legislación alineándose con los estándares de derechos humanos, el MINSAL ha mantenido una gobernanza audista que instrumentaliza la LS como una herramienta técnica accesoria, negando su estatus de derecho cultural fundamental.

Este estudio pone de relieve que la respuesta estatal ante la demanda de autonomía lingüística ha sido tecnocrática y vertical. La insistencia en implementar soluciones de traducción remota sin validación comunitaria, sumada a la validación judicial de la externalización de servicios hacia la atención primaria municipal, ha institucionalizado una inequidad territorial que precariza la atención de alta complejidad.

Para saldar esta deuda histórica, no basta con apelar a la «voluntad política» ni con promulgar nuevas leyes. Se requiere reorientar el modelo de atención desde un enfoque de derechos que asegure el acceso lingüístico como garantía exigible y reconozca a la comunidad sorda como sujeto colectivo, con participación efectiva en las decisiones sanitarias. Esto supone superar el paternalismo rehabilitador e institucionalizar facilitadores interculturales y unidades de referencia especializadas como componentes permanentes de la red hospitalaria. En paralelo, resulta clave fortalecer la formación del personal y la educación pública para desnaturalizar el audismo y consolidar el reconocimiento social de la LS y la cultura sorda. Como proyección, futuras investigaciones deberían evaluar la implementación de estos dispositivos en el sistema público de salud y sus efectos en continuidad del cuidado, seguridad clínica y equidad territorial.
